# Effect of the Matching Circuit on the Electromechanical Characteristics of Sandwiched Piezoelectric Transducers

**DOI:** 10.3390/s17020329

**Published:** 2017-02-09

**Authors:** Shuyu Lin, Jie Xu

**Affiliations:** Shaanxi key Laboratory of Ultrasonics, Shaanxi Normal University, Xi’an 710119, China; jiexu2014@sina.com

**Keywords:** piezoelectric transducer, matching electrical inductor, resonance/anti-resonance frequency, effective electromechanical coupling coefficient, electric quality factor, electro-acoustic efficiency

## Abstract

The input electrical impedance behaves as a capacitive when a piezoelectric transducer is excited near its resonance frequency. In order to increase the energy transmission efficiency, a series or parallel inductor should be used to compensate the capacitive impedance of the piezoelectric transducer. In this paper, the effect of the series matching inductor on the electromechanical characteristics of the piezoelectric transducer is analyzed. The dependency of the resonance/anti-resonance frequency, the effective electromechanical coupling coefficient, the electrical quality factor and the electro-acoustical efficiency on the matching inductor is obtained. It is shown that apart from compensating the capacitive impedance of the piezoelectric transducer, the series matching inductor can also change the electromechanical characteristics of the piezoelectric transducer. When series matching inductor is increased, the resonance frequency is decreased and the anti-resonance unchanged; the effective electromechanical coupling coefficient is increased. For the electrical quality factor and the electroacoustic efficiency, the dependency on the matching inductor is different when the transducer is operated at the resonance and the anti-resonance frequency. The electromechanical characteristics of the piezoelectric transducer with series matching inductor are measured. It is shown that the theoretically predicted relationship between the electromechanical characteristics and the series matching inductor is in good agreement with the experimental results.

## 1. Introduction

Piezoelectric transducers are widely used as emitters, sensors, resonators, filters and actuators in many fields to convert electrical and mechanical energy, such as piezoelectric motors, piezoelectric transformers, ultrasonic cleaning and welding, etc. [1,2,3,4,5,6,7,8,9]. The piezoelectric transducer can be regarded as a two-port network. At the input port of the transducer, an alternating electric signal is applied. By means of the piezoelectric effect, mechanical vibration is produced at the output port of the transducer.

In all kinds of applications of piezoelectric ceramics, piezoelectric sensors are the most widespread and develop rapidly in recent years. Nowadays, piezoelectric sensors are widely used in nondestructive testing and MEMS [Micro-electromechanical Systems] electromechanical coupling systems, such as material and structural health monitoring, including the monitoring of metallic and nonmetallic materials and structures. In piezoelectric sensing applications, electromechanical coupling is very important in electromechanical impedance (EMI) methods and MEMS. In these applications, some new methods and technologies have been developed and new advancements have been achieved [10,11,12,13].

**Figure 1:**
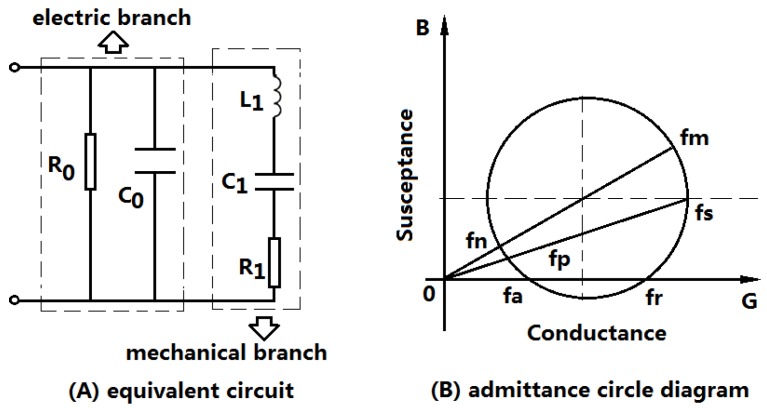
A lumped electromechanical equivalent circuit and admittance circle diagram of a piezoelectric transducer at resonance.

Piezoelectric transducers, including piezoelectric sensors, are almost resonant devices. When they are operated at the resonance frequency, the output mechanical or acoustical power and the energy conversion efficiency reach the maximum value. Near the resonance frequency, the piezoelectric transducer can be represented by a lumped electromechanical equivalent circuit as shown in Figure 1A [14]. The equivalent circuit can be divided into two parts: one is the electric branch, and the other is the mechanical branch. In the figure, $R_{0}$ and $C_{0}$ are the dielectric loss resistance and the clamped capacitance of the piezoelectric transducer; and $R_{1}{,\;L}_{1}$ and $C_{1}$ are the equivalent mechanical resistance, inductor and capacitance arising from the mechanical vibration. $R_{1}$ is composed of two parts, one is the internal mechanical loss of the transducer, the other is the load mechanical resistance.

From the lumped electromechanical equivalent circuit of a piezoelectric transducer, the input electric admittance of a piezoelectric transducer can be obtained. The admittance is frequency-dependent and its circle diagram is illustrated as shown in Figure 1B [15,16]. From the equivalent circuit and the admittance circle diagram, six characteristic frequencies $f_{m}{,f}_{n}{;\;f}_{s}{,f}_{p}{\;\mathrm{and}\;f}_{r}{,f}_{a}$ can be obtained. Where, $f_{m}$ and $f_{n}$ are defined as the minimum and maximum impedance frequencies, which can also be defined as the maximum and minimum admittance frequencies; $f_{s}$ and $f_{p}$ the series and parallel resonance frequencies, and $f_{r}$ and $f_{a}$ the resonance and anti-resonance frequencies. In general cases, these characteristic frequencies are different. However, when the loss resistances and the load are ignored, these frequencies follow the relationship of $f_{m}{=f}_{s}{=f}_{r}{\;\mathrm{and}\;f}_{n}{=f}_{p}{=f}_{a}$. In this case, based on the equivalent circuit, the resonance and anti-resonance frequency of a piezoelectric transducer can be obtained as the following expressions:

(1) $$\omega_{r}{=\omega }_{m}{=\omega }_{s}=\frac{1}{\sqrt{L_{1}C_{1}}}\;$$

(2) $$\omega_{a}{=\omega }_{n}{=\omega }_{p}=\sqrt{\frac{C_{1}{+C}_{0}}{L_{1}C_{1}C_{0}}}\;$$

where $\omega_{m}{=2\pi f}_{m}$, and $\omega_{m}$ represents the angular frequency.

In practical applications, piezoelectric transducers should be excited to vibrate near the resonance frequency corresponding to certain resonant mode. From the equivalent circuit of the piezoelectric transducer, it can be seen that when the transducer is operated rear the resonance frequency, the input electric impedance is capacitive because of the parallel capacitance $C_{0}$.

It is well-known that the electric power into an electric impedance can be obtained by the equation of P=UIcos(θ), where P,U,I,θ are the electric power, the voltage, the current and the phase angle between the voltage and current. The phase angle is determined by the electric impedance. When the impedance is resistive, the phase angle is zero, cos(θ)=1, the electric power reach the maximum value; when the electric impedance is capacitive or inductive, cos(θ)<. In these cases, the electric power is less than that for resistive electric impedance and this can result in a lot of reactive power. For piezoelectric transducers, the electric impedance is capacitive at resonance. It is well known that the reactive power is harmful to the piezoelectric transducer and the electric power source. In order to compensate the capacitive impedance and to reach the maximum electric power, a series or parallel inductor is always added to the piezoelectric transducer to make the electric impedance resistive.

For a series matching inductor, when the piezoelectric transducer is at resonance, the optimal matching inductor $L_{\mathrm{opt}}$ can be obtained from Figure 1A:

(3) $$L_{\mathrm{opt}}=\frac{C_{0}R_{p}^{2}}{{1+(\omega }_{r}C_{0}R_{p}{)}^{2}}\;$$

(4) $$R_{p}=\frac{R_{1}R_{0}}{R_{1}{+R}_{0}}\;$$

It is well known that piezoelectric transducers are resonant devices; their resonance frequencies are dependent on many factors, such as the load variation, the temperature rise and the additional reactive components. When the resonance frequency of the piezoelectric transducer is changed, the tracking of the operating frequency is necessary for the transducer to achieve the optimum working state [17,18,19,20,21,22,23].

In this paper, a matching inductor in series with the piezoelectric transducer is added to compensate for the reactive impedance, and its effect on the electromechanical characteristics of the transducer is analyzed. In several applications of piezoelectric transducers, the input electric power especially for the high power piezoelectric transducer is usually high. For this kind of high power piezoelectric transducer, the input electric power can reach as high as several thousands of watts. In this case, the reactive power can be very high if the capacitive impedance is not compensated. Therefore, the compensation for the capacitive impedance of high power piezoelectric transducers is necessary.

In the following analysis, based on the electromechanical equivalent circuit of a high power sandwiched piezoelectric transducer, which is widely used in high power ultrasonic applications, such as ultrasonic cleaning, ultrasonic welding and ultrasonic machining, the effect of the series matching inductor is theoretically and experimentally studied. Some conclusions are obtained and can be used in the optimization design and analysis of the electric matching of the piezoelectric transducer.

## 2. Theoretical Analysis of a Sandwiched Piezoelectric Transducer with Series Matching Inductor

**Figure 2:**
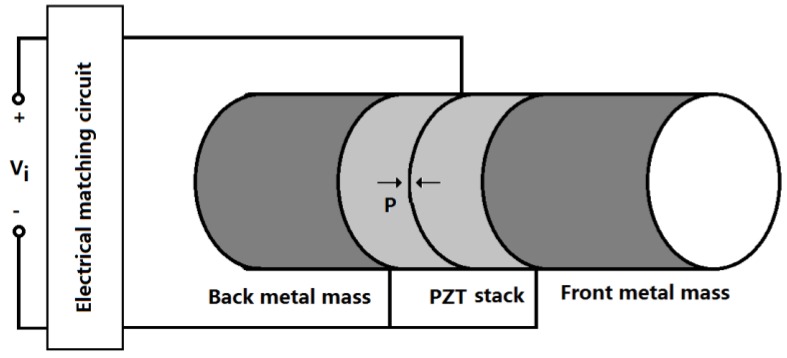
A high power sandwiched piezoelectric transducer with matching circuit.

A high power sandwiched piezoelectric transducer with matching circuit is shown in Figure 2. The sandwiched piezoelectric transducer is composed of the longitudinally polarized piezoelectric stack and the back and front metal masses, which are clamped together by a central pre-stressed high-strength bolt. The matching circuit for the compensation of the capacitive impedance is an inductor which is connected in series with the transducer. The piezoelectric ceramic stack consists of two piezoelectric rings which are longitudinally polarized in opposite direction. In Figure 2, the arrow P represents the polarization direction of the piezoelectric ceramic element. When an external electric signal from a power generator is applied to the piezoelectric ceramic stacks, the transducer will produce vibration in the longitudinal direction because of the inverse piezoelectric effect. When the frequency of the exciting signal is equal to the resonance frequency, the transducer will resonate at a definite vibrational mode, and the vibration of the transducer reaches maximum value.

**Figure 3:**
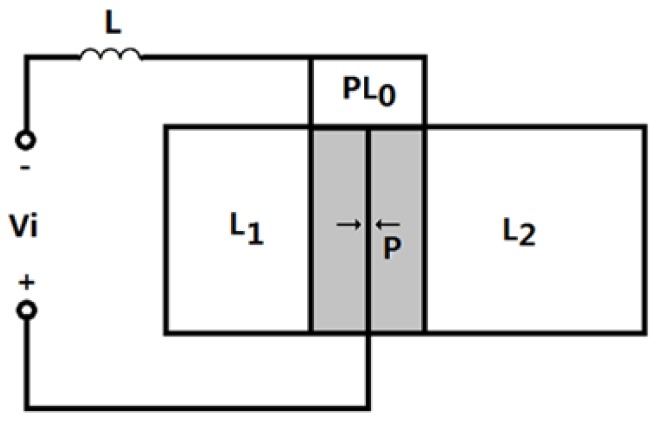
Geometrical sketch of a sandwiched piezoelectric transducer with a series inductor.

The geometrical sketch of a sandwiched piezoelectric transducer with a series matching inductor is shown in Figure 3. In the figure, L represents the matching inductor. The length of the metal masses are $L_{1}$ and $L_{2}$. The length for the piezoelectric ceramic stack is ${\mathrm{pL}}_{0}$, and p is the number of the piezoelectric rings in the piezoelectric stack and it is generally an even number. The radius of the metal mass and the piezoelectric ceramic stack are $R_{1},R_{2}$ and $R_{0}$, respectively. For simplicity, it is assumed that $R_{1}{=R}_{2}{=R}_{0}$.

**Figure 4:**
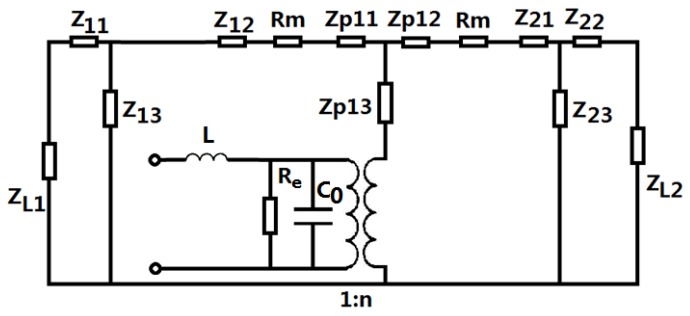
Electro-mechanical equivalent circuit of a sandwiched piezoelectric transducer with a series matching inductor.

Based on the electromechanical equivalent circuit of a longitudinally sandwiched piezoelectric transducer [24,25], the equivalent circuit of a sandwiched piezoelectric transducer with a series inductor is shown in Figure 4. In the figure, $R_{e}$ and $R_{m}$ represent the dielectric losses and the contacting mechanical loss between the contacting surfaces in the transducer. $Z_{L1}$ and $Z_{L2}$ are load mechanical impedances on the mechanical ends. $C_{0}$ and n are the clamped capacitance and electro-mechanical conversion coefficient of the piezoelectric transducers. Their expressions are as follows:

(5) $$C_{0}{=\mathrm{pe}}_{33}^{T}{(1-K}_{33}^{2}{)S}_{0}{/L}_{0}$$

(6) $${n=d}_{33}S_{0}{/(s}_{33}^{E}L_{0})$$

(7) $$R_{e}{=R}_{d}/n$$

where $S_{0}$ is cross sectional areas of the piezoelectric ceramic stack. $S_{0}{=\pi R}_{0}^{2}$, $R_{d}$ is the dielectric loss resistance for each piezoelectric ring in the piezoelectric stack. $\varepsilon_{33}^{T}$, $K_{33}$, $d_{33}$ and $s_{33}^{E}$ are the dielectric constant, the electromechanical coupling coefficient, the piezoelectric constant and the elastic compliance constant of the piezoelectric material. In Figure 4, the expressions for the series and parallel impedances in the equivalent circuit are as follows:

(8) $$Z_{11}{=Z}_{12}{=\mathrm{jZ}}_{1}\tan(\frac{k_{1}L_{1}}{2}),\;Z_{13}=\frac{Z_{1}}{{\mathrm{jsin}(k}_{1}L_{1})}$$

(9) $$Z_{21}{=Z}_{22}{=\mathrm{jZ}}_{2}\tan(\frac{k_{2}L_{2}}{2}),\;Z_{23}=\frac{Z_{2}}{{\mathrm{jsin}(k}_{2}L_{2})}$$

(10) $$Z_{P11}{=Z}_{P12}{=\mathrm{jZ}}_{0}{\tan(\mathrm{pk}}_{0}L_{0}/2),\;Z_{P13}=\frac{Z_{0}}{{\mathrm{jsin}(\mathrm{pk}}_{0}L_{0})}$$

where $Z_{1}{=\rho }_{1}c_{1}S_{1}{,k}_{1}{=\omega /c}_{1}{,c}_{1}{=(E}_{1}{/\rho }_{1}{)}^{1/2}$, $S_{1}{=\pi R}_{1}^{2}$, $Z_{2}{=\rho }_{2}c_{2}S_{2}{,k}_{2}{=\omega /c}_{2}{,c}_{2}{=(E}_{2}{/\rho }_{2}{)}^{1/2}$, $S_{2}{=\pi R}_{2}^{2}$, ω=2πf, $Z_{0}{=\rho }_{0}c_{0}S_{0}{,k}_{0}{=\omega /c}_{0}{,c}_{0}{=[1/(s}_{33}^{E}\rho_{0}{)]}^{1/2}$, $\rho_{1}{,E}_{1}$ and $\rho_{2}{,E}_{2}$ are density and Young’s modulus of the metal masses; $c_{1}{,c}_{2}$ and $c_{0}$ are sound speed of longitudinal vibration. From Figure 4, the input electrical impedance $Z_{i}$ of the piezoelectric transducer can be obtained:

(11) $$Z_{i}=\frac{Z_{m}Z_{c}}{Z_{m}{+n}^{2}Z_{c}}$$

where $Z_{m}$ is the mechanical impedance of the transducer, $Z_{c}$ is the parallel impedance of the clamped capacitance and the dielectric loss resistance. Their expressions are as follows:

(12) $$Z_{m}{=Z}_{p13}+\frac{{(Z}_{p11}{+Z}_{m1}{)(Z}_{p12}{+Z}_{m2})}{Z_{p11}{+Z}_{p12}{+Z}_{m1}{+Z}_{m2}}$$

(13) $$Z_{m1}{=R}_{m}{+Z}_{12}+\frac{Z_{13}{(Z}_{11}{+Z}_{L1})}{Z_{11}{+Z}_{13}{+Z}_{L1}}$$

(14) $$Z_{m2}{=R}_{m}{+Z}_{21}+\frac{Z_{23}{(Z}_{22}{+Z}_{L2})}{Z_{22}{+Z}_{23}{+Z}_{L2}}$$

(15) $$Z_{c}=\frac{R_{e}}{{j\omega C}_{0}R_{e}+1}$$

When a series matching inductor is added, the total input electrical impedance $Z_{\mathrm{is}}$ is:

(16) $$Z_{\mathrm{is}}{=j\omega L+Z}_{i}$$

From Equation (16), the total input electric impedance of the matching inductor and the transducer can be computed and analyzed. When the imaginary part of the input electric impedance is equal to zero, the resonance frequency $f_{r}$ can be found; when the imaginary part of the input electric admittance is equal to zero, the anti-resonance frequency $f_{a}$ can be found. It should be pointed out that, when the mechanical and dielectric losses and the load mechanical impedance are considered, the imaginary part of the input electric impedance or the admittance sometimes are not equal to zero. In these cases, the resonance and the anti-resonance frequency can be approximately substituted by the frequencies at which the input electric impedance reaches the minimum and the maximum values.

Meanwhile, the effective electromechanical coupling coefficient $K_{\mathrm{effc}}$ can be calculated according to the following expression:

(17) $$K_{\mathrm{effc}}=[1-{\left(\frac{f_{r}}{f_{a}}\right)}^{2}{]}^{1/2}$$

It can be seen that the effective electromechanical coupling coefficient $K_{\mathrm{effc}}$ is determined by the resonance and anti-resonance frequencies of the piezoelectric transducer, which depend on the material, the geometrical dimensions and structure, and the electrical and mechanical loads. It is obvious that the effective electromechanical coupling coefficient is different from the electromechanical coupling coefficient of piezoelectric material, which is only determined by the piezoelectric material, and therefore it is defined as the piezoelectric material parameter, such as $K_{31}$, $K_{33}$ and $K_{t}$.

The electric quality factor $Q_{e}$ can be obtained as the following equation:

(18) Qe=Abs[Im[Zis]Re[Zis]]

where Re[Zis] and Im[Zis] are the real and imaginary parts of the input electric impedance. It is well known that the electro-acoustical efficiency of a piezoelectric transducer is an important parameter; it describes the conversion capacity of the electric power to acoustical power. It is defined as the ratio of the output acoustical power over the input electric power:

(19) $$\eta_{1}=\frac{P_{1}}{P_{i}}{,\eta }_{2}=\frac{P_{2}}{P_{i}},\eta =\frac{P_{1}{+P}_{2}}{P_{i}}$$

where $\eta_{1}$ and $\eta_{2}$ are the electro-acoustical efficiencies when the piezoelectric transducer radiates ultrasonic wave from the back and front mechanical end, respectively, and η is the total electro-acoustical efficiencies when the transducer radiates ultrasonic wave from both the two ends. $P_{1}$ and $P_{2}$ are the radiated acoustical powers from the front and back mechanical ends, and $P_{i}$ is the input electric power.

From the equivalent circuit and the above analysis, the detailed expressions for the electro-acoustical efficiencies can be obtained as:

(20) $$\eta_{1}=\frac{P_{1}}{P_{i}}=\frac{Z_{L1}}{Z_{\mathrm{is}}}\cdot \frac{1}{A^{2}}$$

(21) $$\eta_{2}=\frac{P_{2}}{P_{i}}=\frac{Z_{L2}}{Z_{\mathrm{is}}}\cdot \frac{1}{B^{2}}$$

(22) $$\eta =\frac{P_{1}{+P}_{2}}{P_{i}}=\frac{1}{Z_{\mathrm{is}}}\cdot (\frac{Z_{L1}}{A^{2}}+\frac{Z_{L2}}{B^{2}})$$

where A and B are two introduced constants, their expressions are as follows,

(23) $$A=n\cdot \frac{Z_{c}+\frac{Z_{m}}{n^{2}}}{Z_{c}}\cdot \frac{Z_{p11}{+Z}_{p12}{+Z}_{m1}{+Z}_{m2}}{Z_{p12}{+Z}_{m2}}\cdot \frac{Z_{11}{+Z}_{13}{+Z}_{L1}}{Z_{13}}$$

(24) $$B=n\cdot \frac{Z_{c}+\frac{Z_{m}}{n^{2}}}{Z_{c}}\cdot \frac{Z_{p11}{+Z}_{p12}{+Z}_{m1}{+Z}_{m2}}{Z_{p11}{+Z}_{m1}}\cdot \frac{Z_{22}{+Z}_{23}{+Z}_{L2}}{Z_{23}}$$

In the above analysis, the analytical expressions for the input electric impedance, the effective electromechanical coupling coefficient, the electric quality factor and the electro-acoustical efficiency are obtained. It is obvious that these parameters are dependent on the transducer material, the geometrical dimensions, the frequency and the series matching inductor.

## 3. Effect of the Series Matching Inductor on the Electromechanical Characteristics of a Piezoelectric Transducers

In this section, the effect of the series matching inductor is theoretically analyzed by using a numerical method. The material and geometrical dimensions of the piezoelectric transducer are kept unchanged. The material for the metal masses is aluminum alloy, its standard material parameters are used and as follows: $\rho_{1}{=\;\rho }_{2}{=\;2790\;\mathrm{kg}/m}^{3}$, $E_{1}{=\;E}_{2}=\;7.15\times 10^{10}{\;N/m}^{2}$. The piezoelectric material is an equivalent of PZT-4, and the related material parameters are: $\rho_{0}{=\;7500\;\mathrm{kg}/m}^{3}$, $s_{33}^{E}=\;15.5\cdot 10^{-12}{\;m}^{2}/N$, $K_{33}=\;0.7$, $d_{33}=\;496\cdot 10^{-12}\;C/N$, $\varepsilon_{0}=\;8.8542\cdot 10^{-12}\;F/m$, $\varepsilon_{33}^{T}{/\varepsilon }_{0}=\;1300$. The geometrical dimensions of the transducer are $R_{1}{=\;R}_{2}=\;19.5\;\mathrm{mm}$, $R_{0}=19.0\;\mathrm{mm}$, $L_{1}{=\;L}_{2}=\;56\;\mathrm{mm}$, $L_{0}=\;5\;\mathrm{mm}$, $Z_{L1}{=\;Z}_{L2}=\;200\;\mathrm{ohm}$, $R_{e}=\;12,000\;\mathrm{ohm}$, $R_{m}=\;100\;\mathrm{ohm}$, p = 2.

### 3.1. Effect of the Series Matching Inductor on the Frequency Response

**Figure 5:**
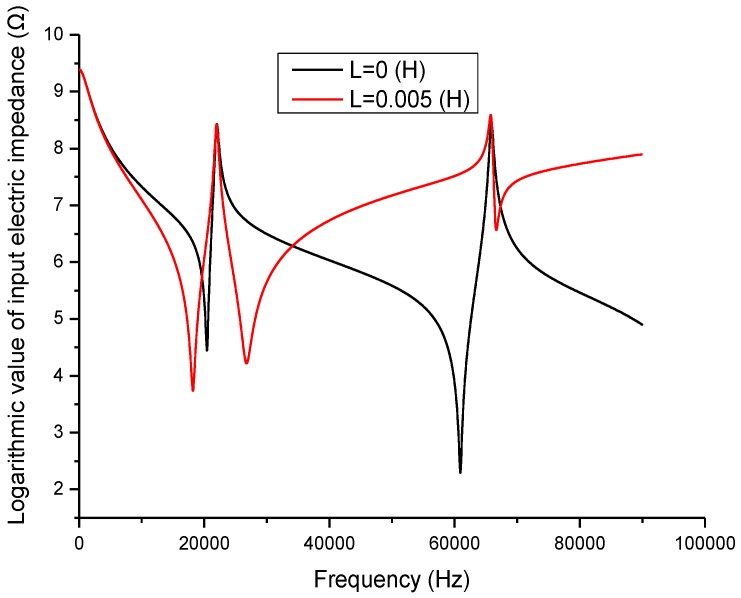
Frequency response of the input electric impedance.

**Figure 6:**
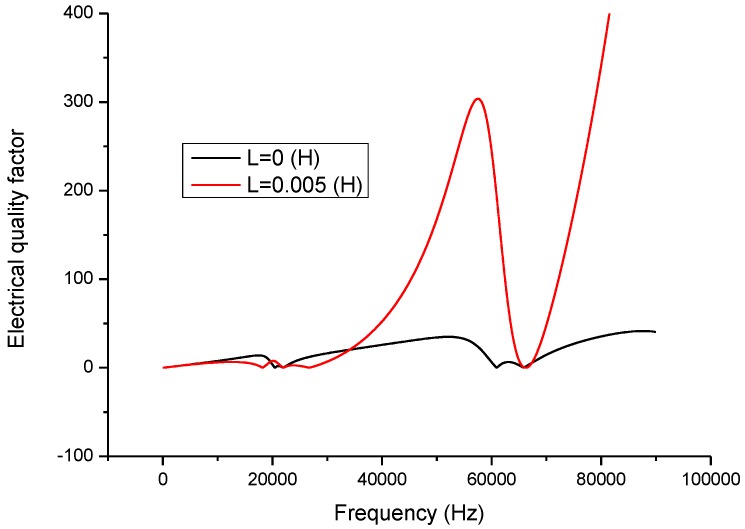
Frequency response of the electric quality factor.

**Figure 7:**
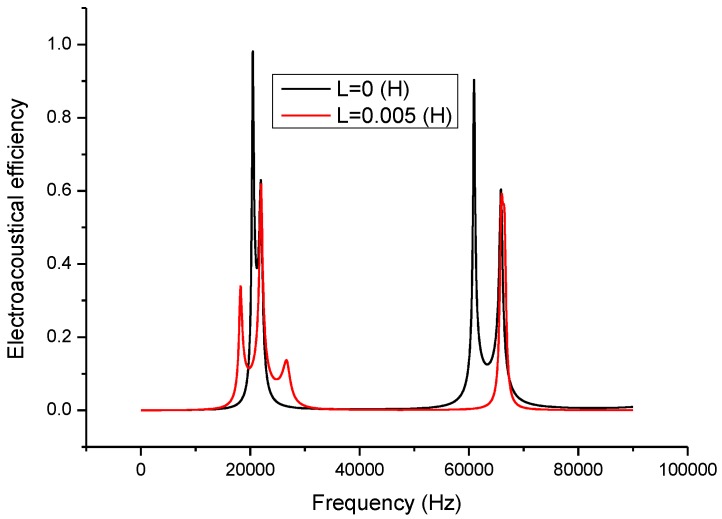
Frequency response of the electro-acoustical efficiency.

Electromechanical impedance technique is now widely used in analyzing the electromechanical characteristics of piezoelectric devices and systems [26,27,28,29]. In the present study, this technique is adopted to analyze the effect of the inductor on the piezoelectric transducer. When a series matching inductor is changed, the frequency response of the input electric impedance, the electric quality factor and the electro-acoustical efficiency of a piezoelectric transducer with series matching inductor are obtained as shown in Figure 5, Figure 6 and Figure 7. In the figures, the frequency range extends to the second longitudinal mode of the piezoelectric transducer.

Figure 5 illustrates the relationship between the input electric impedance and the working frequency. In the figure, the frequencies corresponding to the minimum and maximum impedance are the resonance and anti-resonance frequencies. It can be seen that when an inductor is connected in series with the piezoelectric transducer, the resonance frequency is decreased, while the anti-resonance frequency is almost unchanged.

Figure 6 describes the relationship between the electric quality factor and frequency. At the resonance and anti-resonance frequencies, the electric quality factor has maximum values. When there is no series matching inductor, the electric quality factor at the resonance frequency is larger than at the anti-resonance frequency. When a series matching inductor is added, the electric quality factor at the resonance frequency is smaller than at the anti-resonance frequency.

Figure 7 illustrates the relationship between the electro-acoustic efficiency and the frequency. It can be seen that when a series matching inductor is added, the electro-acoustical efficiency at resonance frequency is decreased and remains unchanged at anti-resonance frequency. On the other hand, when there is no series matching inductor, the electro-acoustic efficiency at resonance frequency is larger than at anti-resonance frequency. When a series matching inductor is added, the electro-acoustic efficiency at resonance frequency is lower than at anti-resonance frequency.

### 3.2. Dependency of the Electromechanical Parameters on the Series Matching Inductor

**Figure 8:**
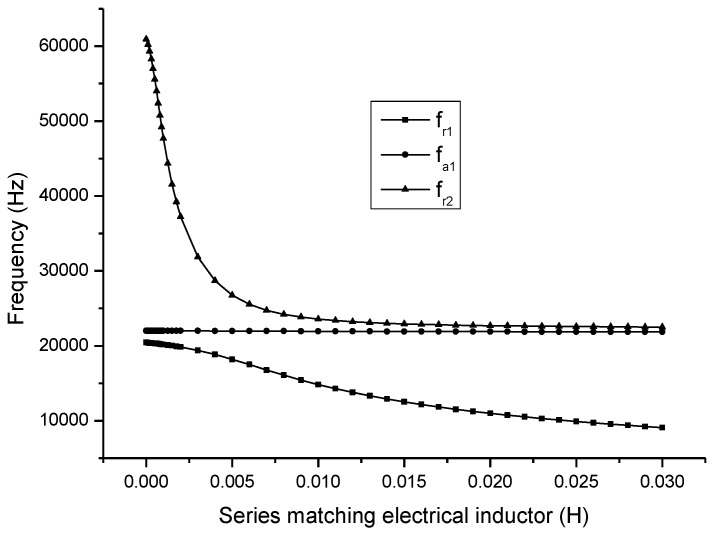
Theoretical relationship between the resonance/anti-resonance frequency and the series matching inductor.

The relationship between the electromechanical parameters and the series matching inductor are obtained as shown in Figure 8.

In Figure 8, $f_{r1}$ and $f_{r2}$ are the first and the second resonance frequency, $f_{a1}$ is the first anti-resonance frequency. It can be seen that when the series matching inductor is increased, the resonance frequency is decreased and the anti-resonance frequency is almost unchanged.

**Figure 9:**
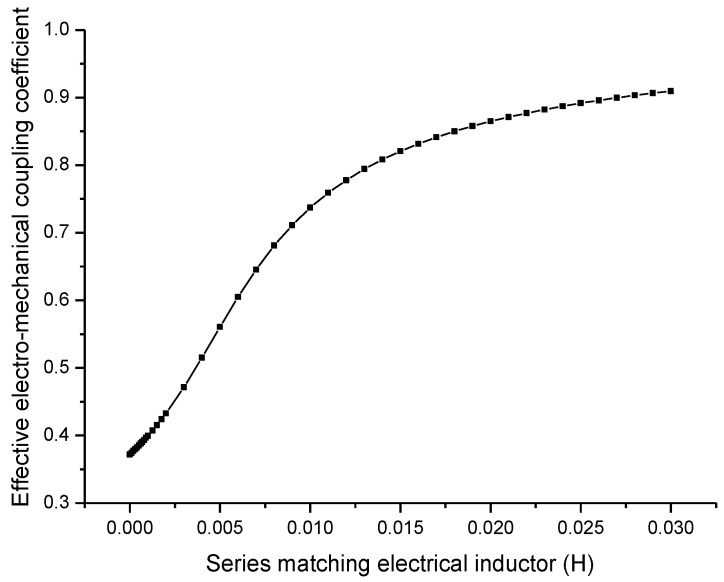
Dependency of the effective electromechanical coupling coefficient on the series matching inductor.

From Figure 9, it can be seen that when the series matching inductor is increased, the effective electromechanical coupling coefficient is increased. It should be noted that Figure 9 is a general relationship between the series inductor and the effective electromechanical coupling coefficient. For any practical transducers, the capacitive impedance at resonance is definite, in order to compensate this capacitive impedance, there is an optimal value for the series matching inductor. In general cases, this optimal series matching inductor is not very large and, therefore, the effective electromechanical coupling coefficient is always less than one. The optimal series matching inductor depends on the resonance frequency and the parallel capacitance of the piezoelectric transducer.

**Figure 10:**
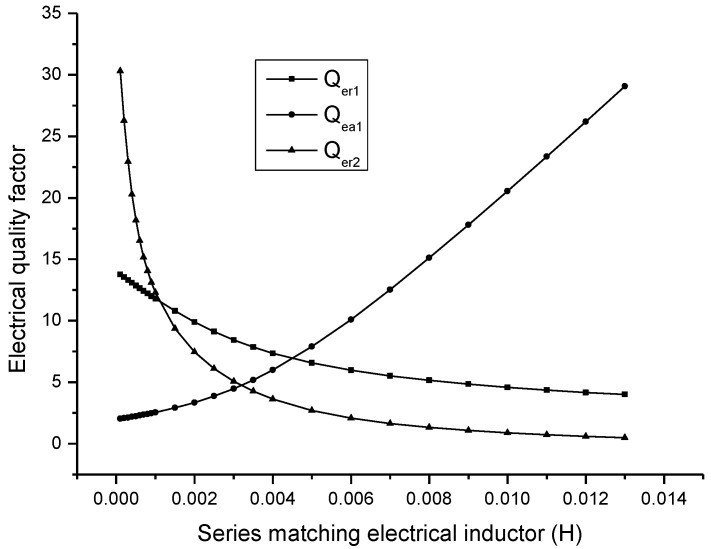
Theoretical relationship between the electric quality factor and the series matching inductor.

In Figure 10, $Q_{\mathrm{er}1}$ and $Q_{\mathrm{er}2}$ are the electric quality factors corresponding to the first and the second resonance frequency, and $Q_{\mathrm{ea}1}$ is the electric quality factor corresponding to the first anti-resonance frequency. It is shown that when the series matching inductor is increased, the electric quality factor corresponding to resonance frequency is decreased, while the electric quality factor corresponding to anti-resonance frequency is increased.

**Figure 11:**
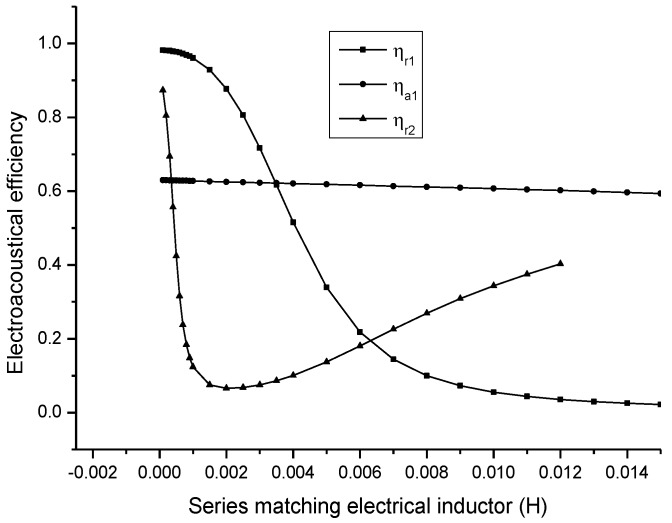
Dependency of the electro-acoustic efficiency on the series matching inductor.

In Figure 11, $\eta_{r1}$ and $\eta_{r2}$ are the electro-acoustical efficiency corresponding to the first and the second resonance frequency, and $\eta_{a1}$ is the electro-acoustical efficiency corresponding to the first anti-resonance frequency. It is shown that when the series matching inductor is increased, the electro-acoustical efficiency corresponding to the first resonance frequency is decreased; the electro-acoustical efficiency corresponding to anti-resonance frequency is unchanged. The electro-acoustical efficiency corresponding to the second resonance frequency has a minimum value.

From the above analyses, it can be seen that the relationship between the effective electromechanical coupling coefficient, the electro-acoustic efficiency, the electric quality factor and the series matching inductor is different.

The electro-acoustic efficiency at resonance frequency is decreased when the series matching inductor is increased. The electro-acoustic efficiency at anti-resonance frequency is basically unchanged when the series matching inductor is increased. When the series matching inductor is increased, the effective electromechanical coupling coefficient is increased. The electric quality factor at resonance frequency is decreased with the increase of the series matching inductor. It is obvious that the variation tendency of the electro-acoustic efficiency and the electric quality factor with the series matching inductor is contradictory to that of the effective electro-mechanical coupling coefficient with the series matching inductor. Therefore, in the practical design of the series matching inductor, a compromise between the electro-acoustic efficiency, the electric quality factor and the effective electro-mechanical coupling coefficient should be made.

## 4. Experiments

**Figure 12:**
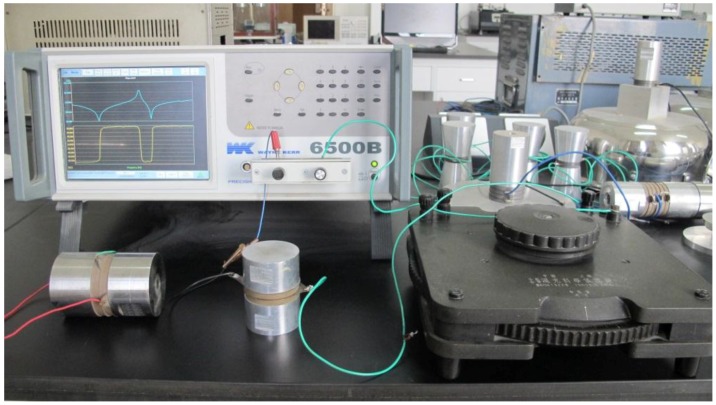
Experimental set-up for the measurement of the frequency response.

**Figure 13:**
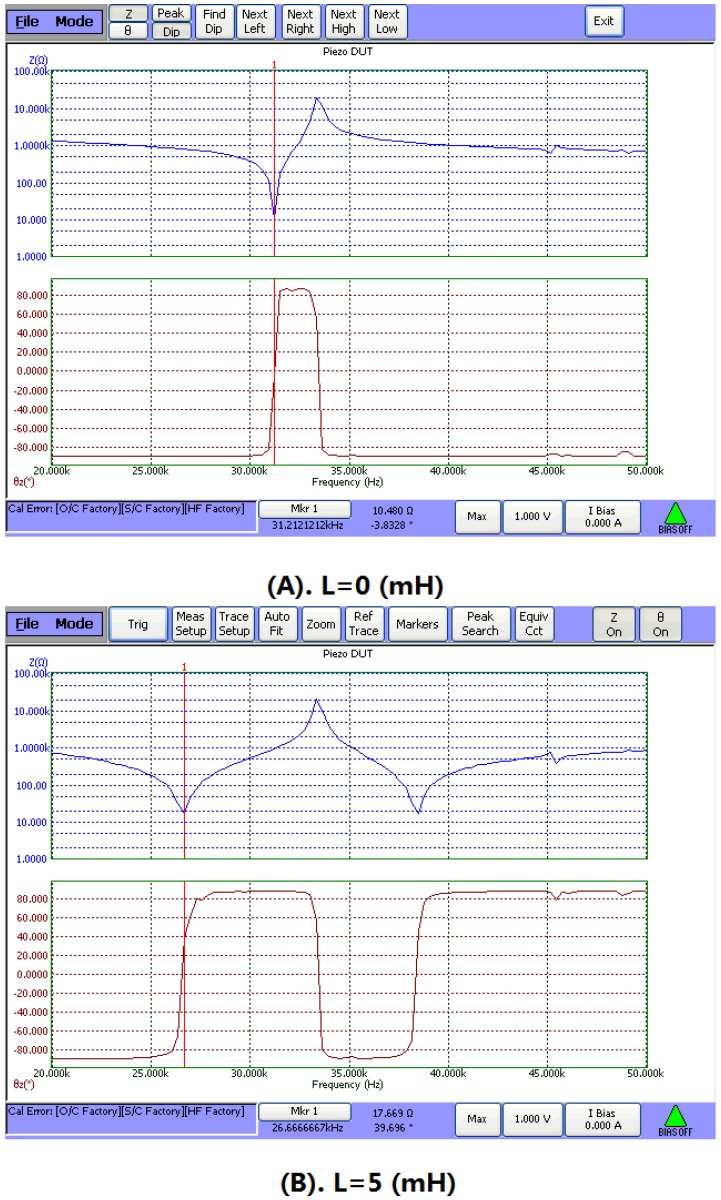
Measured frequency response of the input electric impedance of a sandwich piezoelectric transducer. (**A**) L = 0 (mH); (**B**) L = 5 (mH).

In this section, the effect of the series matching inductor on the sandwiched piezoelectric transducer is experimentally studied. Two sandwiched piezoelectric transducers are made and their resonance and anti-resonance frequency are obtained by measuring the frequency response of the input electric impedance using 6500B Precision Impedance Analyzer. Figure 12 is the experimental set-up and Figure 13 illustrates the measured frequency response of a sandwiched piezoelectric transducer. It can be seen that when a series inductance is added, the resonance frequency is decreased while the anti-resonance frequency is almost unchanged.

The measured results are listed in Table 1. In the table, f_r_, f_a_, K_effc_ and f_mr_, f_ma_, K_meffc_ are the theoretical and measured resonance/anti-resonance frequencies and effective electromechanical coupling coefficient.

From the above results, it can be seen that the theoretical dependency of the resonance/anti-resonance frequencies and the effective electromechanical coupling coefficient on the series matching inductor is in agreement with the experimental results.

## 5. Conclusions

The effect of a series matching inductor on the sandwiched piezoelectric power transducer is studied. The dependency of the resonance/anti-resonance frequency, the electric quality factor, the effective electromechanical coupling coefficient and the electro-acoustic efficiency on the series matching inductor is analyzed. To sum up the above analysis, the following conclusions can be drawn:

1. When a matching inductor is connected in series with a sandwiched piezoelectric transducer, the resonance frequency is decreased and the anti-resonance frequency is almost unchanged; the effective electromechanical coupling coefficient is increased.
2. Without a series matching inductor, the electro-acoustical efficiency at resonance frequency is larger than that at anti-resonance frequency. When a series matching inductor is added, the electro-acoustical efficiency at resonance frequency is decreased and remains unchanged at anti-resonance frequency, and the electro-acoustical efficiency at resonance frequency is lower than at anti-resonance frequency.
3. When the series matching inductor is increased, the electric quality factor corresponding to resonance frequency is decreased, while the electric quality factor corresponding to anti-resonance frequency is increased.

## Figures and Tables

**Table 1:** Experimental resonance/anti-resonance frequencies and effective electromechanical coupling coefficient of the piezoelectric transducers.

No.	L (mH)	f_r_ (Hz)	f_a_ (Hz)	f_mr_ (Hz)	f_ma_ (Hz)	K_effc_	K_meffc_
1	0	33805	39570	31212	33333	0.52	0.35
	5	17823	39554	26607	33333	0.89	0.60
	10	12990	39539	20606	33333	0.94	0.79
2	0	20585	28706	18182	20000	0.70	0.42
	5	14699	28677	17727	20000	0.86	0.46
	10	11560	28650	16364	20000	0.91	0.57
